# Update on recent preclinical and clinical studies of T790M mutant-specific irreversible epidermal growth factor receptor tyrosine kinase inhibitors

**DOI:** 10.1186/s12929-016-0305-9

**Published:** 2016-12-03

**Authors:** Bin-Chi Liao, Chia-Chi Lin, Jih-Hsiang Lee, James Chih-Hsin Yang

**Affiliations:** 1 Department of Oncology, National Taiwan University Hospital, 7, Chung-Shan South Road, Taipei, 100 Taiwan; 2 National Taiwan University Cancer Center, College of Medicine, National Taiwan University, Taipei, Taiwan; 3 Graduate Institute of Clinical Medicine, College of Medicine, National Taiwan University, Taipei, Taiwan; 4 Graduate Institute of Oncology, College of Medicine, National Taiwan University, Taipei, Taiwan; 5 Department of Urology, College of Medicine, National Taiwan University, Taipei, Taiwan; 6 Department of Medical Research, National Taiwan University Hospital, Taipei, Taiwan

**Keywords:** Non-small cell lung cancer, Epidermal growth factor receptor, Tyrosine kinase inhibitor, T790M mutation, Osimertinib, Rociletinib, Olmutinib, EGF816, ASP8273

## Abstract

The first- and second-generation epidermal growth factor receptor tyrosine kinase inhibitors (1/2G EGFR-TKIs) gefitinib, erlotinib, and afatinib have all been approved as standard first-line treatments for advanced *EGFR* mutation-positive non-small cell lung cancer. The third-generation (3G) EGFR-TKIs have been developed to overcome the *EGFR* T790M mutation, which is the most common mechanism of acquired resistance to 1/2G EGFR-TKI treatment. This resistance mutation develops in half of the patients who respond to 1/2G EGFR-TKI therapy. The structures of the novel 3G EGFR-TKIs are different from those of 1/2G EGFR-TKIs. Particularly, 3G EGFR-TKIs have lower affinity to wild-type EGFR, and are therefore associated with lower rates of skin and gastrointestinal toxicities. However, many of the adverse events (AEs) that are observed in patients receiving 3G EGFR-TKIs have not been observed in patients receiving 1/2G EGFR-TKIs. Although preclinical studies have revealed many possible mechanisms for these AEs, the causes of some AEs remain unknown. Many mechanisms of resistance to 3G EGFR-TKI therapy have also been reported. Here, we have reviewed the recent clinical and preclinical developments related to novel 3G EGFR-TKIs, including osimertinib, rociletinib, olmutinib, EGF816, and ASP8273.

## Background

The first-generation reversible epidermal growth factor receptor tyrosine kinase inhibitors (1G EGFR-TKIs) gefitinib and erlotinib are both quinazoline derivatives, as is the second-generation (2G) irreversible EGFR-TKI afatinib. These drugs are effective for treating advanced *EGFR* mutation-positive non-small cell lung cancer (NSCLC), especially in patients who harbor *EGFR* exon 21 L858R mutation (*EGFR* ^L858R^) or exon 19 deletions (*EGFR* ^del19^). Accordingly, all of these drugs are currently standard first-line therapies for these patients [1–6]. However, these drugs also inhibit wild-type EGFR (EGFR^wt^), and diarrhea and skin acne/rash are common adverse events (AEs). After a period of 9 to 11 months of effective treatment, acquired resistance to 1G/2G EGFR-TKIs inevitably ensues. About 50–60% of the cases of acquired resistance are attributable to the *EGFR* T790M mutation, which is the substitution of threonine with methionine at amino acid position 790, *EGFR* ^T790M^ [7–12]. Novel third-generation (3G) EGFR-TKIs were designed to overcome this major mechanism of resistance while also having less capacity to inhibit EGFR^wt^, thereby minimizing the AEs that are seen in 1G/2G EGFR-TKI therapy. Here, we have reviewed the recent preclinical and clinical developments related to 3G EGFR-TKIs with a special focus on the unusual AEs that are associated with these novel drugs. We have also reviewed the mechanisms of acquired resistance to these drugs and the possible solutions by which these resistance mechanisms may be overcome.

A literature review of clinical studies published between January 2013 and June 2016 was conducted using PubMed and MEDLINE, with the entry keywords ‘non-small cell lung cancer,’ ‘epidermal growth factor receptor T790M mutation,’ ‘osimertinib,’ ‘rociletinib,’ ‘olmutinib,’ ‘EGF816,’ and ‘ASP8273.’ We also performed a manual search of the abstracts presented at major oncology meetings.

## Main text of the review

### Osimertinib

Osimertinib (AZD9291) is a mono-anilino-pyrimidine compound that irreversibly targets tumors harboring *EGFR* ^L858R^, *EGFR* ^del19^, and *EGFR* ^T790M^, while having little effect on EGFR^wt^. This compound makes a covalent bond with cysteine residue in position 797 of EGFR (Cys797), and also has activity against other kinases that harbor cysteine residue in the analogous kinase domain, such as ErBB2, ErBB4, and BLK (BLK proto-oncogene, Src family tyrosine kinase; previous name: B lymphoid tyrosine kinase). Like *EGFR* ^T790M^, insulin receptor and insulin-like growth factor 1 receptor also have methionine gatekeeper in their kinase domains. Nonetheless, osimertinib does not have significant activity against either of these receptors [13, 14].

In the first phase I/II clinical study of orally administered osimertinib (AURA), 80 mg/day was chosen as the dose for subsequent phase II or III studies, even though a true dose-limiting toxicity was not observed at this dose level [15]. In a pooled analysis of two studies (AURA phase II extension cohort and AURA 2), outcomes were examined for patients who had *EGFR* mutation-positive NSCLC, whose disease had progressed following previous EGFR-TKI therapy, whose tumors harbored *EGFR* ^T790M^, and who had been treated with osimertinib at 80 mg/day. Among the 397 evaluable patients, the confirmed objective response rate (ORR) was 66% and the disease control rate (DCR) was 91%. The median progression-free survival (PFS) was 11.0 months (*n* = 411). The observed treatment-related AEs are listed in Table 1, and only < 1% of the patients developed grade ≥ 3 skin rash or diarrhea. Three percent of the patients developed interstitial lung disease (ILD) and QT interval corrected for heart rate (QTc) prolongation while hyperglycemia developed in less than 1% of the patients [16]. Some grade ≥ 3 laboratory abnormalities, such as neutropenia (3.4%), lymphopenia (3.3%), thrombocytopenia (1.2%), and hyponatremia (3.4%) were also reported [17].

**Table 1:** Selected clinical efficacy reports of third-generation EGFR-TKIs

Drug name	Number	Patient group	Dose	ORR	PFS	AEs (% total, % ≥ grade 3)^a^	ILD (%)	Distinct AEs
Osimertinib	411	EGFR-TKI pretreated advanced *EGFR* ^T790M^–positive NSCLC	80 mg/day	66% (95% CI, 61–71)	11.0 months (95% CI 9.6–12.4)	Skin rash (41, < 1), diarrhea (38, < 1), dry skin (30, 0), QTc prolongation (3, 1)	3	Neutropenia, lymphopenia, thrombocytopenia, hyponatremia, QTc prolongation
Rociletinib	548	EGFR-TKI pretreated advanced *EGFR* ^T790M^–positive NSCLC	500–750 mg twice per day	33.9% (95% CI, 29.5–38.5)	5.7 months (95% CI 4.2–6.2) at 500 mg twice a day	Hyperglycemia (65.2, 35.2), skin rash (11.7, 0.4), diarrhea (57.5, 4.6), QTc prolongation (30.1, 10.2)	2.4	Hyperglycemia, cataract, QTc prolongation, pancreatitis
Olmutinib	76	EGFR-TKI pretreated advanced *EGFR* ^T790M^–positive NSCLC	800 mg/day	54%	6.9 months(95% CI 5.36–9.49)	Diarrhea (59, 0), pruritus (42, 1), rash (41, 5), nausea (39, 0), Palmar-plantar erythrodysesthesia syndrome (30, 4)	1	Palmar-plantar erythrodysesthesia syndrome
EGF816	152	Advanced *EGFR* mutation-positive NSCLC^b^	75–350 mg/day	46.9% (95% CI, 38.7–55.3)	9.7 months (95% CI 7.3–11.1)	Skin rash (53.9, 16.4), diarrhea (36.8, 2), pruritus (34.2, NA), dry skin (25.0, NA), stomatitis (24.3, 2.0)	0.7	Distinct skin rash, hepatitis B virus reactivation, increased serum lipase level
ASP8273	63	Advanced *EGFR* mutation-positive NSCLC (92% harbored *EGFR* ^T790M^)	300 mg/day	30% (95% CI, 19.2–43.0)	6.0 months (95% CI 4.1–9.8)	Diarrhea (48, 2), nausea (27, 0), paresthesia (14, 0), vomiting (13, 0), dizziness (11, 0), and hyponatremia (19, 13)	0	Hyponatremia, paresthesia

*Abbreviations*: *EGFR* epidermal growth factor receptor, *TKI* tyrosine kinase inhibitor, *ORR* objective response rate, *PFS* progression-free survival, *AE* adverse event, *ILD* interstitial lung disease, *NSCLC* non-small cell lung cancer, *CI* confidence interval, *QTc* QT interval corrected for heart rate, *NA* not available

^a^For each AE, reported values in this column are (the percent of patients receiving the therapy who experience the AE, the percent of patients receiving the therapy who experienced the AE at grade ≥ 3)

^b^Including patients harbored sensitizing *EGFR* mutations following EGFR-TKI therapy (regardless of *EGFR* ^T790M^ status), *EGFR* exon 20 insertion or deletion, de novo T790M mutation, and patients with treatment-naïve advanced *EGFR* mutation-positive NSCLC

In November 2015, osimertinib received US Food and Drug Administration (FDA) approval for EGFR–TKI-pretreated metastatic *EGFR* ^T790M^-positive NSCLC, as did the companion diagnostic test (cobas® EGFR Mutation Test v2) that is used to detect tumor *EGFR* ^T790M^. By July 2016, osimertinib had also received approvals in the European Union, Japan, South Korea, Canada, Switzerland, Israel, and Mexico. A confirmatory phase III study (AURA 3, ClinicalTrials.gov, NCT02151981) is comparing osimertinib with platinum-based chemotherapy in patients who have advanced *EGFR* mutation-positive NSCLC, whose disease progressed following first-line EGFR-TKI therapy, and whose tumors harbor *EGFR* ^T790M^. This study has completed patient accrual and is ongoing.

Because osimertinib has activity against sensitizing *EGFR* mutations and is associated with reduced skin rash and diarrhea AEs, it has also been tested as a first-line treatment for metastatic *EGFR* mutation-positive NSCLC. Two expansion cohorts in the AURA study enrolled patients with metastatic *EGFR* mutation-positive NSCLC and tested the safety and efficacy of first-line osimertinib monotherapy. Osimertinib monotherapy was tested at 80 and 160 mg/day, and a total of 60 patients were enrolled (30 at each dose level). The ORR was 67% at 80 mg/day and 87% at 160 mg/day. The DCR was 93% at 80 mg/day and 100% at 160 mg/day. For 80 mg/day, the median PFS had not been reached at the time of the data cutoff for the analysis, and the 18-month progression-free survival rate was 57%. For 160 mg/day, the median PFS was 19.3 months, and the 18-month progression-free survival rate was 53%. All grades skin rash and diarrhea developed in 70 and 87% of patients receiving 80 mg/day, respectively, as well as 60 and 80% of patients receiving 160 mg/day, respectively. Three percent and 7% of patients developed grade ≥ 3 skin rash and grade ≥ 3 diarrhea at 160 mg/day, respectively. ILD and QTc prolongation developed in 10 and 0% of patients receiving 80 mg/day, as well as 7 and 10% of patients receiving 160 mg/day, respectively [18]. A phase III randomized study (FLAURA study, ClinicalTrials.gov, NCT02296125) is comparing osimertinib with gefitinib or erlotinib as first-line therapies in patients with advanced *EGFR* mutation-positive NSCLC. The study has completed patient accrual and is ongoing.

Osimertinib showed clinical activity for brain metastases in the AURA and AURA 2 studies [19]. Leptomeningeal metastasis is another detrimental complication of advanced *EGFR* mutation-positive NSCLC [20]. A phase I study (BLOOM study, ClinicalTrials.gov, NCT02228369) is ongoing to test the safety and efficacy of osimertinib monotherapy against brain and leptomeningeal metastasis. In a preliminary report, osimertinib at 160 mg/day showed promising activity against leptomeningeal metastasis [21].

Combination therapy is another treatment strategy for conferring better anti-tumor activity. In the TATTON study (ClinicalTrials.gov, NCT02143466), osimertinib was combined with either MET inhibitor (AZD6094, savolitinib), MEK inhibitor (selumetinib), or anti-PD-L1 monoclonal antibody (MEDI4736, durvalumab) [22]. However, a preliminary report showed that the incidence of ILD was high in the osimertinib plus durvalumab arm. A phase III study of osimertinib plus durvalumab versus osimertinib monotherapy (CAURAL study, ClinicalTrials.gov, NCT02454933), also showed a high incidence of ILD in the combination arm, and the development of osimertinib plus durvalumab combination therapy was therefore discontinued [23]. Investigations of other combination therapies are ongoing, such as for osimertinib in combination with necitumumab, ramucirumab, or bevacizumab (ClinicalTrials.gov, NCT02496663, 02789345, and 02803203). In addition to metastatic disease, clinical trials of osimertinib monotherapy for *EGFR* mutation-positive NSCLC are also ongoing in the adjuvant setting (ADAURA study, ClinicalTrials.gov, NCT02511106).

Another important issue in the development of 3G EGFR-TKIs is the application of liquid biopsies to detect *EGFR* ^T790M^ in blood or urine samples [24–27]. Plasma samples were collected in the AURA study, and the cell-free plasma DNA was genotyped using the beads, emulsions, amplification, and magnetics (BEAMing) digital polymerase chain reaction technique (Sysmex Inostics, Inc., Mundelein, IL, USA) [25, 28]. The plasma-based sensitivity for detecting *EGFR* ^T790M^ was 70%. The ORR and median PFS were similar in patients with *EGFR* ^T790M^-positive plasma and those with *EGFR* ^T790M^-positive tissue, which was defined as the gold standard (ORR: 63 vs. 62%; median PFS: 9.7 vs. 9.7 months). The authors concluded that patients with *EGFR* ^T790M^-positive plasma could avoid tumor re-biopsy for *EGFR* ^T790M^ testing, while those with *EGFR* ^T790M^-negative plasma should undergo tumor re-biopsy [29]. Mechanisms of resistance to osimertinib therapy have also been reported. In a preclinical cell line study, acquired *EGFR* C797S mutation (the substitution of cysteine with serine at amino acid position 797, *EGFR* ^C797S^) was identified as a mechanism of resistance to osimertinib therapy. This is understandable because osimertinib forms a key covalent bond with EGFR at the position of the noted cysteine residue. Resistant cells that contain sensitizing mutations (*EGFR* ^L858R/C797S^ and *EGFR* ^del19/C797S^) retain sensitivity to quinazoline-based 1G/2G EGFR-TKIs, such as gefitinib and afatinib, but are resistant to these drugs in the presence of *EGFR* ^T790M^ (*EGFR* ^L858R/T790M/C797S^ and *EGFR* ^del19/T790M/C797S^). However, resistant cells that harbor *EGFR* ^L858R/T790M/C797S^ remain partially sensitive to cetuximab because of the disruption of EGFR dimerization [30]. Another preclinical study also provided evidence that *EGFR* ^C797S^ is a resistance mechanism [31]. Furthermore, the authors of that study demonstrated that, if *EGFR* ^C797S^ occurred *in trans* (on a different allele) from *EGFR* ^T790M^, then the resistant cells were sensitive to a combination of 1G/3G EGFR-TKIs. In contrast, if the two mutations occurred *in cis* (on the same allele), then the cells were resistant to the combination therapy. Other mechanisms of resistance to osimertinib therapy have also been identified in cell line studies, including *NRAS* ^E63K^ mutation and gains of copy number for wild-type *NRAS* and wild-type *KRAS*. Combination therapy with osimertinib and selumetinib prevented and delayed the developments of resistance [32].

In the clinical setting, *EGFR* ^C797S^ was first described in a patient who developed acquired resistance to osimertinib therapy. In this case, *EGFR* ^C797S^ was detected in a cell-free plasma DNA analysis that was performed using next-generation sequencing. A subsequent study collected plasma samples from 15 patients who received osimertinib therapy and had pre-existing plasma *EGFR* ^T790M^ that was detected using droplet digital polymerase chain reaction. Upon developing resistance, 6 (40%) patients had *EGFR* ^del19/T790M/C797S^, 5 (33%) patients had *EGFR* ^T790M^ alone, and *EGFR* ^T790M^ was no longer detectable in 4 (27%) patients who retained prior sensitizing mutations [33]. Mechanistically, *EGFR* ^C797S^ parallels the acquired Bruton tyrosine kinase (BTK) C481S mutation, which is observed in patients with chronic lymphocytic leukemia who develop acquired resistance to therapy with ibrutinib, an irreversible BTK inhibitor. Provided in combination with cetuximab, a novel *EGFR*–resistance-mutation selective allosteric inhibitor (EAI045) has been observed to be effective in a mouse model of NSCLC harboring *EGFR* ^L858R/T790M/C797S^. Cetuximab blocks the dimerization of EGFR and renders the kinase susceptible to this allosteric agent [34]. *EGFR* ^C797S^ was also detected in tumor re-biopsy samples from a patient who developed acquired resistance to osimertinib [35]. Several other acquired resistance mechanisms were reported in patients who experienced disease progression on osimertinib therapy: acquired *EGFR* ^L718Q^, small cell transformation, *MET* amplification, *HER2* amplification, *BRAF* ^V600E^ mutation, *PIK3CA* ^E545K^ mutation, loss of *EGFR* ^T790M^ plus alternative pathway activation, and EGFR ligand-dependent activation [36–41]. In a case report, a patient who developed *MET* amplification responded to therapy with crizotinib, an ALK and MET inhibitor [36].

### Rociletinib

Rociletinib (CO-1686) is a 2,4-disubstituted pyrimidine compound that irreversibly targets tumors harboring *EGFR* ^L858R^, *EGFR* ^del19^, and *EGFR* ^T790M^, while having little effect on EGFR^wt^. There is a meta-acrylamide that points to Cys797 and forms the covalent bond. This compound also has activities against other kinases, such as FAK, CHK2, ErBB4, and JAK3 [42, 43]. A metabolite of rociletinib, M502, has potency against insulin receptor and insulin-like growth factor 1 receptor, which may lead to the AE of hyperglycemia [44].

An early-phase clinical study (TIGER X, ClinicalTrials.gov, NCT01526928) determined that orally administered rociletinib at 500 mg twice per day was the recommended dose for subsequent clinical study [45, 46]. An update report of the 208 patients who received rociletinib at 500 mg twice per day disclosed that any grade of hyperglycemia, diarrhea, nausea, and QTc prolongation developed in 57.2, 56.7, 43.8, and 26.4% of the patients, respectively. Of the patients, 28.8 and 7.7% developed grade ≥ 3 hyperglycemia and grade ≥ 3 QTc prolongation, respectively. ILD was observed in 0.5% of the patients at this dose level [47]. An unexpected AE of sudden-onset cataract developed in 21 of 40 patients (53%) who were treated with rociletinib in a single-hospital study, and most of these patients required surgical repair [48]. In the TIGER-X study, the overall incidence of cataract was 9.1% in patients who received rociletinib therapy at dose levels of 500–750 mg twice per day [47]. Additionally, this drug has limited activity against central nervous system metastases [49, 50]. In the first report of the TIGER-X study, the ORR of rociletinib in patients who harbored *EGFR* ^T790M^ was 59% [45]; however, those patients who achieved partial response (PR) as their best response did not have a subsequent confirmed PR at least 4 weeks apart, per the RECIST criteria (version 1.1) [51]. Rociletinib did not receive accelerated approval by the US FDA. An official report of the TIGER-X study has updated the confirmed ORR to 33.9% for the efficacy population of 443 patients who received rociletinib at dose levels of 500–750 mg twice per day, and who had centrally confirmed *EGFR* ^T790M^-positive NSCLC. The PFS was 5.7 months in 208 patients who received rociletinib therapy at 500 mg twice per day. The safety profile of 548 patients from the study is shown in Table 1 [47]. A biomarker study that used tissue, plasma (BEAMing), and urine specimens (Trovera Quantitative NGS assay, Trovagene, San Diego, CA, USA) to detect *EGFR* ^T790M^ showed sensitivities of 80.9 and 81.1% based on plasma and urine, respectively. The confirmed ORRs in patients with *EGFR* ^T790M^-positive tissue, plasma, and urine were 33.9, 32.1, and 36.7%, respectively. Patients with M1a/M0 intrathoracic disease had lower plasma sensitivity than did patients with M1b distant metastatic disease (56.8 vs. 88.4%, *p* < .001) [52].

Ongoing clinical studies include the TIGER-2 study (ClinicalTrials.gov, NCT02147990), which is a phase II study that seeks to test the safety and efficacy of rociletinib as a second-line treatment for advanced *EGFR* mutation-positive NSCLC, following progression on prior EGFR-TKI therapy. Additionally, the TIGER-3 study (ClinicalTrials.gov, NCT02322281) is a phase III study that seeks to compare rociletinib with single-agent cytotoxic chemotherapy as a third-line or later treatment for advanced *EGFR* mutation-positive NSCLC in patients for whom EGFR-TKI and platinum-doublet therapy have failed. Patients with *EGFR* ^T790M^-positive and -negative disease were both eligible for the two aforementioned studies.

Rociletinib is also being tested in the first-line setting in TIGER-1 (ClinicalTrials.gov, NCT02186301), which is a randomized phase II/III study that is comparing rociletinib with erlotinib as first-line treatments for advanced *EGFR* mutation-positive NSCLC. Studies of combination therapies are ongoing, such as for rociletinib in combination with trametinib (a MEK inhibitor) (ClinicalTrials.gov, NCT02580708) or atezolizumab (MPDL3280A, an anti-PD-L1 monoclonal antibody) (ClinicalTrials.gov, NCT02630186). However, in May 2016, Clovis Oncology, Inc. announced that it had terminated enrollment in all ongoing sponsored studies of rociletinib and withdrawn its Marketing Authorization Application for rociletinib from European regulatory authorities [53].

A preclinical study identified epithelial-mesenchymal transition as a possible mechanisms of resistance to rociletinib therapy that can be overcome by AKT inhibitors [43]. A cell line study identified *MET* amplification with or without *EGFR* ^T790M^ as a mechanism of resistance to CNX-2006 (tool compound of rociletinib). In a cell line that had *MET* amplification without *EGFR* ^T790M^, MET tyrosine kinase inhibitor was able to overcome the resistance by itself, suggesting an oncogenic shift from *EGFR* to *MET*. The authors described this phenomenon as “oncogene swap” [54].

In clinical studies, mechanisms of resistance to rociletinib were identified by using plasma circulating tumor DNA profiling (Cancer Personalized Profiling by Deep Sequencing, CAPP-Seq). *MET* copy number gain is the most frequent mechanism, and was observed in 43 (26%) patients in a recent study [55]. In this study, rociletinib-resistant xenografts also developed *MET* amplification that could be overcome using crizotinib therapy. *EGFR* ^C797S^ and novel *EGFR* ^L798I^ were each identified in one patient. Other mechanisms involving *MET*, *EGFR*, *PIK3CA*, *ERRB2*, *KRAS,* and *RB1* were also described. Nineteen percent of the patients displayed resistance mechanisms that affected multiple genes, a finding that further emphasizes the importance of tumor heterogeneity [55]. In another study, tumor re-biopsy after progression on rociletinib therapy showed that loss of *EGFR* ^T790M^ plus *EGFR* amplification and small cell transformation were mechanisms of resistance. The authors emphasized the concept of tumor heterogeneity, in which *EGFR* ^T790M^-positive and -negative tumor cells may coexist in a tumor before rociletinib therapy, and for which it is not sufficient to target only one mechanism of resistance [56]. A short clinical report demonstrated that, following the development of resistance to rociletinib, some patients still responded to osimertinib therapy [57].

### Olmutinib

Olmutinib (BI 1482694 / HM61713) is an irreversible kinase inhibitor that binds to a cysteine residue near the kinase domain. Olmutinib shows activities against cell lines and xenograft tumors harboring *EGFR* ^L858R/T790M^ and *EGFR* ^del19^, while having little effect on cell lines with EGFR^wt^ [58].

In the first phase I/II study (ClinicalTrials.gov, NCT01588145) conducted in South Korea, orally administered olmutinib at 800 mg/day was identified as the recommended dose for subsequent studies. In that study, 76 *EGFR* ^T790M^-positive patients received olmutinib therapy at a dose of 800 mg/day, and the median PFS was 6.9 months. The confirmed ORR was 54% among 70 evaluable patients, and activity against central nervous system metastases was also observed [59]. The treatment-related AEs from this study are listed in Table 1. One patient experienced ILD and discontinued therapy, but there was no AE of hyperglycemia [59].

In December 2015, olmutinib was granted the breakthrough therapy designation for NSCLC by the US FDA. In May 2016, it was approved in South Korea for advanced *EGFR* ^T790M^-positive NSCLC patients who were pretreated with EGFR-TKIs [60]. Regarding the future clinical development of olmutinib, the phase II ELUXA 1 study (ClinicalTrials.gov, NCT02485652) is recruiting patients with advanced *EGFR* ^T790M^-positive NSCLC after prior EGFR-TKI therapy. Other global clinical trial plans for olmutinib are available online [61]. Regarding first-line treatment, a phase II study of first-line olmutinib for advanced *EGFR* mutation-positive NSCLC was conducted in South Korea (ClinicalTrials.gov, NCT02444819).

Acquired resistance to olmutinib was reported in a patient who developed *EGFR* ^C797S^ after a period of effective olmutinib therapy [62].

### EGF816

EGF816 irreversibly targets EGFR by forming a covalent bond to Cys797. Preclinical data show activities against cell lines and xenograft models harboring *EGFR* ^L858R/T790M^, *EGFR* ^del19/T790M^, *EGFR* ^del19^, and *EGFR* ^L858R^, while having little effect on cell lines harboring EGFR^wt^ [63]. In an early-phase clinical study (ClinicalTrials.gov, NCT02108964), 152 patients were enrolled to receive orally administered EGF816 at 75–350 mg/day. The common AEs in this study are shown in Table 1. Grade ≥ 3 AEs included rash (16.4%), anemia (2.6%), urticaria (2.6%), diarrhea (2.0%), and fatigue (2.0%). The type and distribution of the skin rash were different from the acneiform rash that is observed in patients treated with 1G/2G EGFR-TKIs. Two and 2 patients developed hepatitis B virus reactivation and increased lipase level, respectively [64]. The confirmed ORR and DCR among 147 evaluable patients were 46.9 and 87.1%, respectively. The estimated PFS across all dose levels was 9.7 months [64]. In a preclinical study, EGF816 also showed activity against *EGFR* exon 20 insertion. Therefore, the early-phase study also enrolled patients with tumors harboring this genetic alteration [63]. A study of the combination therapy of EGF816 and nivolumab (an anti-PD-1 monoclonal antibody) is ongoing (ClinicalTrials.gov, NCT02323126).

A preclinical study demonstrated several mechanism of resistance to EGF816, including *EGFR* ^C797S^, *MET* amplification, and epithelial-mesenchymal transition. Dual inhibition of EGFR and cMET with EGF816 and INC280 (a MET inhibitor) can overcome this resistance mechanism [63]. A phase Ib/II study of EGF816 and INC280 combination therapy for advanced *EGFR* mutation-positive NSCLC is in progress (ClinicalTrials.gov, NCT02335944).

### ASP8273

ASP8273 is a mutant-selective irreversible EGFR inhibitor that targets EGFR by forming a covalent bond to Cys797. Preclinical data show activities against cell lines and xenograft models harboring *EGFR* ^L858R/T790M^, *EGFR* ^del19/T790M^, *EGFR* ^del19^, and *EGFR* ^L858R^, while having little effect on cell lines harboring EGFR^wt^ [65, 66]. In a first-in-human phase I/II study (ClinicalTrials.gov, NCT02192697) conducted in Japan, ASP8273 has been well tolerated across multiple dose levels. Common treatment-related AEs have included diarrhea, vomiting, nausea, hyponatremia, increased serum alanine transaminase level, and thrombocytopenia. A few patients have experienced rash, QTc prolongation, and ILD-like events. Three hundred mg/day was chosen as the recommended dose for subsequent phase II studies [67]. An update report showed a preliminary ORR (including both confirmed and unconfirmed response) of 64% in 70 *EGFR* ^T790M^-positive patients treated with ASP8273 at 300 mg/day [68]. This study is ongoing in Japan, South Korea, and Taiwan.

In another phase I study that was conducted in the US (ClinicalTrials.gov, NCT02113813), orally administered ASP8273 at 300 mg/day was chosen as the recommended dose for subsequent phase II studies. A total of 110 patients were enrolled and 63 patients received treatment at the dose of 300 mg/day. Ninety-two percent of these 63 patients harbored *EGFR* ^T790M^. The confirmed ORR was 30% and the median PFS was 6.0 months [69]. Common treatment-related AEs are listed in Table 1. Grade ≥ 3 AEs included diarrhea (2%) and hyponatremia (13%). One subject developed grade 1 hyperglycemia, and no patient developed ILD or QTc prolongation [69].

ASP8273 was mainly developed as a first-line treatment for advanced *EGFR* mutation-positive NSCLC in a phase II study in Japan (ClinicalTrials.gov, NCT02500927). The global randomized phase III SOLAR study (ClinicalTrials.gov, NCT02588261) is comparing ASP8273 with gefitinib or erlotinib as first-line treatments for advanced *EGFR* mutation-positive NSCLC.

### Others

Other third-generation EGFR-TKIs are currently under early clinical development, including PF-06747775 (Pfizer, ClinicalTrials.gov, NCT02349633), avitinib (Hangzhou ACEA Pharmaceutical Research Co., Ltd., ClinicalTrials.gov, NCT02330367 and NCT02274337), brigatinib (ARIAD, AP26113, ALK and EGFR inhibitor, ClinicalTrials.gov, NCT01449461), and TAS-121 (Taiho Pharma) [70–75].

## Discussion

The *EGFR* ^T790M^ mechanism of acquired resistance to 1G EGFR-TKIs was first reported in 2005 [9, 10]. *EGFR* ^T790M^ is also the major mechanism of acquired resistance to therapy using the 2G EGFR-TKI afatinib [7]. The prognostic role of acquired *EGFR* ^T790M^ was controversial before the era of 3G EGFR-TKIs [12, 26, 76, 77]. Indeed, there were many different theories about the development of drug-resistant cells, such as the pre-existence theory and evolution from drug-tolerant cells. The drug-tolerant state may provide an environment in which heterogeneous drug-resistance mechanisms can be developed [78–81].

3G EGFR-TKIs have been developed to address the *EGFR* ^T790M^ resistance mechanism, and phase I dose finding studies of these 3G EGFR-TKIs have been conducted almost exclusively for patients with the *EGFR* ^T790M^ mutation. These “atypical” phase I studies have several characteristics in common: First, these studies (for example AURA and TIGER-X) enrolled large numbers of patients in their phase I components. Second, given the effectiveness of the 3G EGFR-TKIs, these studies expanded patient numbers at the same dose level and were extended to the phase II component within the same overall study, in order to accelerate accruals. Third, because of the relatively low rates of treatment-related toxicities that were observed, the recommended phase II/III doses are not necessary the true maximum tolerated doses. Fourth, because of the complexities of the trial designs, caution is warranted when interpreting their clinical outcomes. The application of liquid biopsies (plasma or urine) using various modalities is one of the attractive features of these studies, and the concordance rates between tissue and liquid biopsy results have been high [29, 52]. Nonetheless, the current diagnostic standard for *EGFR* ^T790M^ is still tumor re-biopsy for tissue sampling, and the role of liquid biopsy should be explored further in prospective trials [82, 83].

The resistance mechanisms to 3G EGFR-TKIs were discovered either via preclinical investigations, clinical liquid biopsy, or tumor re-biopsy. Some of the resistance mechanisms revealed in preclinical studies were later confirmed in clinical studies. *EGFR* ^C797S^ and *MET* amplification/copy number gain are the most important of the mechanisms of resistance. Novel treatment strategies are under development to overcome these resistance mechanisms [30, 31, 33, 36–40, 54–57, 62]. In light of tumor heterogeneity, *EGFR* ^T790M^-positive and -negative cells may coexist in the same tumor, or may occur at different sites in the same patient. 3G EGFR-TKI-based combination therapies that seek to overcome the bypass pathways are a reasonable strategy for addressing the coexistence of *EGFR* ^T790M^-positive and -negative cells [39, 56, 84].

Some distinct AEs have been observed in patients who received 3G EGFR-TKI therapy. For example, neutropenia, lymphopenia, thrombocytopenia, and QTc prolongation have been observed in patients receiving osimertinib; hyperglycemia and cataract have been observed in patients receiving rociletinib; distinct skin rash and hepatitis B virus reactivation have been observed in patients receiving EGF816; and hyponatremia and paresthesia have been observed in patients receiving ASP8273. Although some of these AEs are attributable to off-target effects, the causes of others remain unknown. These rare and unexpected AEs were prudently observed by the investigators in these early-phase studies, and information on their occurrence spread to all collaborators in a timely manner; the importance of tight global collaboration cannot be overemphasized [85]. The incidences of ILD were low in the clinical trials, although they excluded patients with a history of ILD.

Given their high inhibitory activity against sensitizing *EGFR* mutations, some 3G EGFR-TKIs are under development as first-line treatments that are being compared with 1G/2G EGFR-TKIs (FLAURA [osimertinib], TIGER-1 [rociletinib], ELUXA 1 [olmutinib], SOLAR [ASP8273] studies). Investigators should be more cautious of ILD and other AEs associated with these therapies. When administered as a first-line treatment, the combination therapy of osimertinib and durvalumab induced a high incidence of ILD, which serves as an important reminder that the same drugs can have unexpected consequences when provided in different clinical settings and combinations [23].

In summary, the development of 3G EGFR-TKIs is encouraging because they have often shown noteworthy effectiveness and reduced rates of classic AEs, such as diarrhea and skin rash. The application of these drugs in different settings and combinations should be explored in the future.

## Conclusions

Osimertinib therapy is the current standard of care for patients with *EGFR* mutation-positive NSCLC who developed acquired *EGFR* ^T790M^ after prior EGFR-TKI therapy. Other 3G EGFR-TKIs are currently under development. Liquid biopsy is being investigated as a means of both detecting plasma/urine *EGFR* ^T790M^ prior to 3G EGFR-TKI therapy and identifying mechanisms of resistance to 3G EGFR-TKI therapy. Various combination therapies that incorporate a 3G EGFR-TKI aim to prolong the benefits of 3G EGFR-TKIs and/or overcome resistance mechanisms. Many clinical trials of first-line 3G EGFR-TKI therapies are in progress, and their findings may ultimately change the paradigm of standard first-line treatment for *EGFR* mutation-positive NSCLC.

## Abbreviations

1G: First-generation
2G: Second-generation
3G: Third-generation
AEs: Adverse events
DCR: Disease control rate
EGFR: Epidermal growth factor receptor
*EGFR*^C797S^: *EGFR* C797S mutation
*EGFR*^del19^: *EGFR* exon 19 deletions
*EGFR*^L858R^: *EGFR* exon 21 L858R mutation
*EGFR*^T790M^: *EGFR* T790M mutation
EGFR^wt^: Wild-type EGFR
EGFR-TKIs: EGFR tyrosine kinase inhibitors
FDA: Food and Drug Administration
ILD: Interstitial lung disease
NSCLC: Non-small cell lung cancer
ORR: Objective response rate
PFS: Progression-free survival
PR: Partial response
QTc: QT interval corrected for heart rate
